# Supplementary material for: Cooperative transport mechanism of human monocarboxylate transporter 2
Source: Nat Commun. 2020 May 15;11:2429. doi: 10.1038/s41467-020-16334-1 (PMC7228944; doi:10.1038/s41467-020-16334-1)
Supplement: Supplementary file 1 — Supplementary Information [file 41467_2020_16334_MOESM1_ESM.pdf]

Supplementary information of “Cooperative transport mechanism of human monocarboxylate transporter 2”

Bo Zhang[1#], Qiuheng Jin[1#], Lizhen Xu[2#], Ningning Li[3#], Ying Meng[4], Shenghai Chang[5,6], Xiang Zheng[7], Jiangqin Wang[5,8], Yuan Chen[7], Dante Neculai[4], Ning Gao[3], Xiaokang Zhang[9*], Fan Yang[2*], Jiangtao Guo[5,8*], Sheng Ye[9,1*]

[1] Life Sciences Institute, Zhejiang University, Hangzhou 310058, China;

[2] Department of Biophysics and Kidney Disease Center, First Affiliated Hospital, Institute of Neuroscience, NHC and CAMS Key Laboratory of Medical Neurobiology, Zhejiang University School of Medicine, Hangzhou 310058, China;

[3] State Key Laboratory of Membrane Biology, Peking-Tsinghua Center for Life Sciences, School of Life Sciences, Peking University, Beijing, China;

[4]Department of Cell Biology, School of Basic Medical Sciences, Zhejiang University, Hangzhou, Zhejiang, P.R. China;

[5] Department of Biophysics, Department of Pathology of Sir Run Run Shaw Hospital, Zhejiang University School of Medicine, Hangzhou 310058, China;

[6] Center of Cryo Electron Microscopy, Zhejiang University School of Medicine, Hangzhou 310058, China;

[7] The State Key Laboratory of Subtropical Silviculture, Zhejiang A & F University, 666 Wusu street, Lin’an 311300, China;

[8] Department of Biophysics, Institute of Neuroscience, NHC and CAMS Key Laboratory of Medical Neurobiology, Zhejiang University School of Medicine,

Hangzhou, 310058, China;

[9] Tianjin Key Laboratory of Function and Application of Biological Macromolecular Structures, School of Life Sciences, Tianjin University, 92 Weijin Road, Nankai District, Tianjin 300072, China;

[#]These authors contributed equally.

Correspondence and requests for materials should be addressed to S.Y. (sye@tju.edu.cn), J.G. (jiangtaoguo@zju.edu.cn), F.Y. (fanyanga@zju.edu.cn), and X.Z. (xzhang1965@zju.edu.cn).

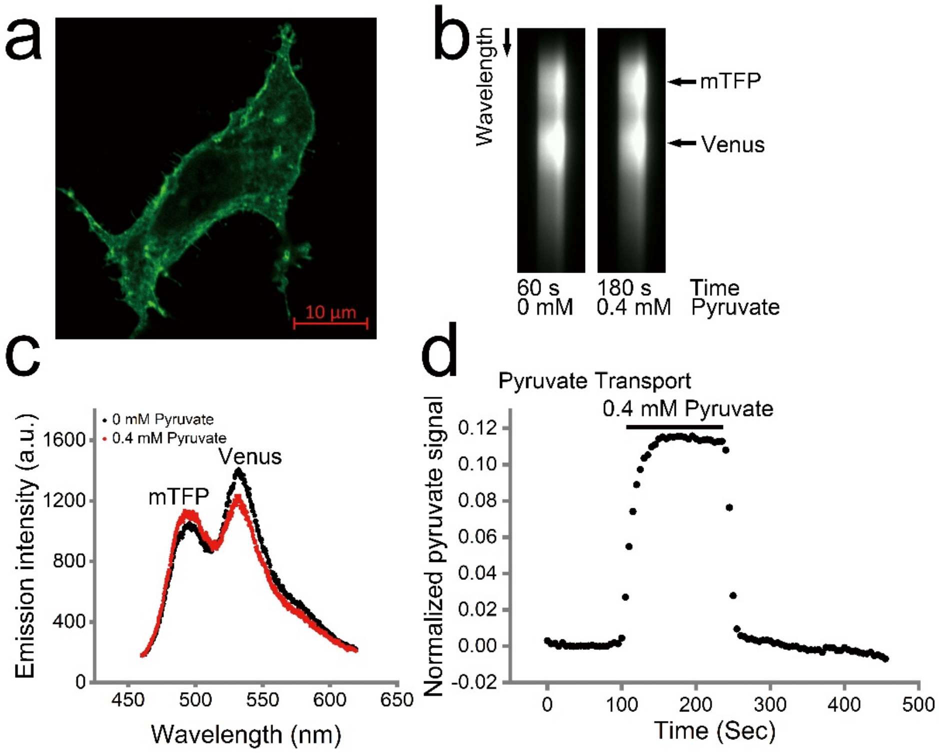

Supplementary Figure 1 | Spectral analysis of pyruvate flux in HEK293T cells expressing WT MCT2 and pyronic. a, Spectral images at two selected time frames from Supplementary Video 1. At the time point of 60 sec, the cell was exposed in the buffer without pyruvate. Whereas at the time point of 180 sec, the cell was exposed in the buffer with 0.4 mM pyruvate. Scale bar, 10 µm. **b,** Emission spectra (excited by a 420/20 nm band-pass filter) above 460 nm at two selected frames. 0 mM pyruvate: the cell was exposed in the buffer without pyruvate. 0.4 mM pyruvate: the cell was exposed in the buffer with 0.4 mM pyruvate. **c,** A cell was bathed in a buffer containing 0 mM pyruvate, was then exposed to a buffer containing 0.4 mM pyruvate, and finally back to a buffer without pyruvate. Representative trace depicting fluorescence ratio (cytosolic pyruvate signal). Baseline drift was corrected with initial 50 sec of trace prior to addition of pyruvate (normalized pyruvate signal).

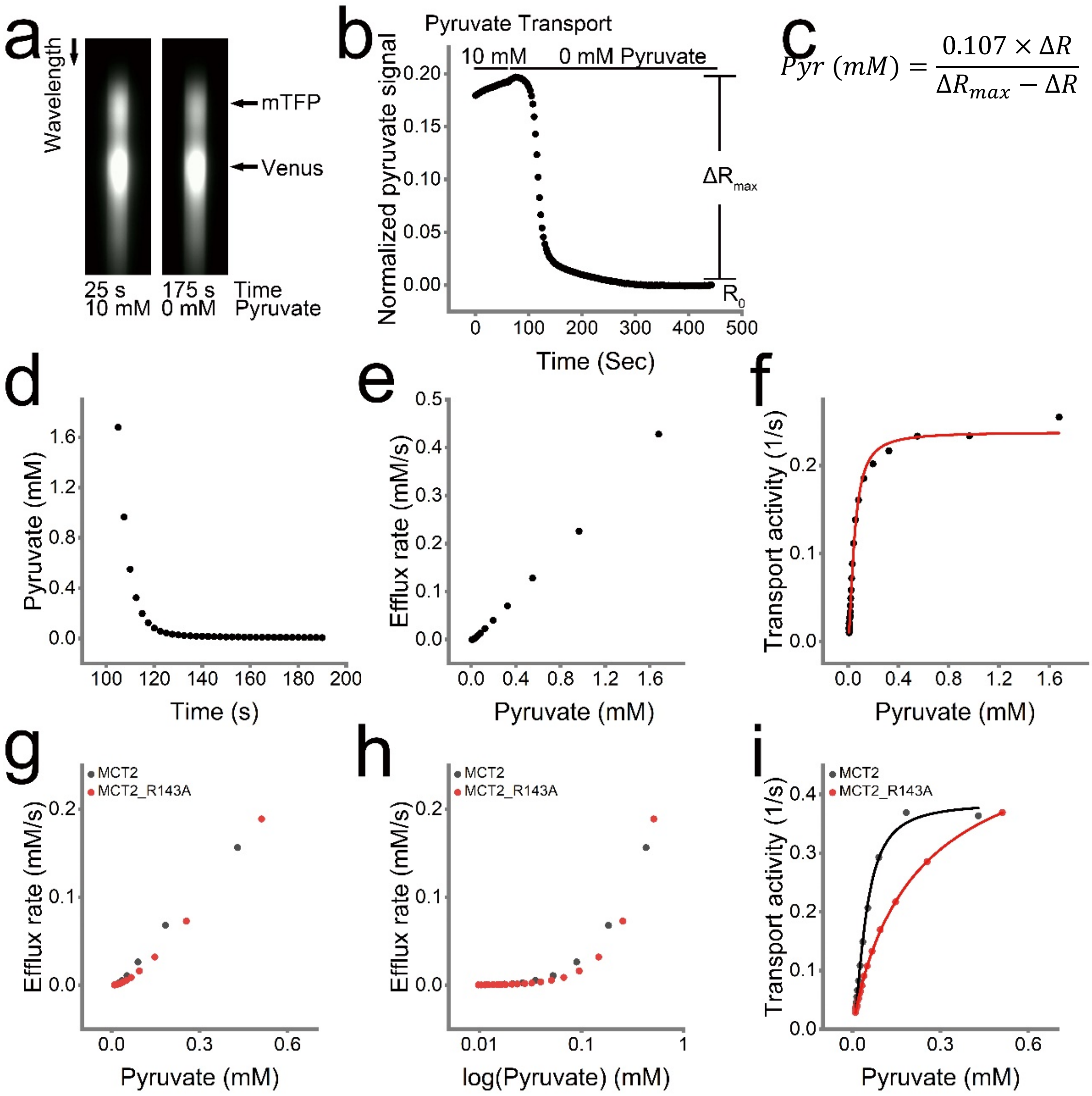

Supplementary Figure 2 | Spectral analysis of pyruvate flux in HEK293T cells expressing MCT2 and pyronic, a, Spectral images at two selected time frames from Supplementary Video 2. At the time point of 25 sec, the cell was exposed in a buffer containing 10 mM pyruvate, whereas at the time point of 175 sec, the cell was exposed in a buffer without pyruvate. **b,** Representative trace depicting normalized cytosolic pyruvate signal of a cell switching from a buffer containing 10 mM pyruvate to a buffer without pyruvate. The trace shows the fluorescence ratio, where R_0 is the

fluorescence ratio of pyronic in the absence of pyruvate and ΔRmax is the difference between R0 and the maximun ratio estimated in 10 mM pyruvate. **c**, Formula used to convert FRET signal to intracellular pyruvate concentration. ΔRmax is described in **b**. ΔR is the value of the ratio at each time point minus R0. **d-i**, Plot of **d**, shows the intracellular pyruvate concentration as a function of time. Plots of **e**, **g**, **h**, pyruvate efflux rate or **f**, **i**, transport activity of **e**, **f**, WT MCT2 or **g**, **h**, **i**, R143A MCT2 measured at an extracellular pyruvate concentration of 0 mM as a function of intracellular pyruvate concentration for MCT2 at an extracellular pH 7.4.

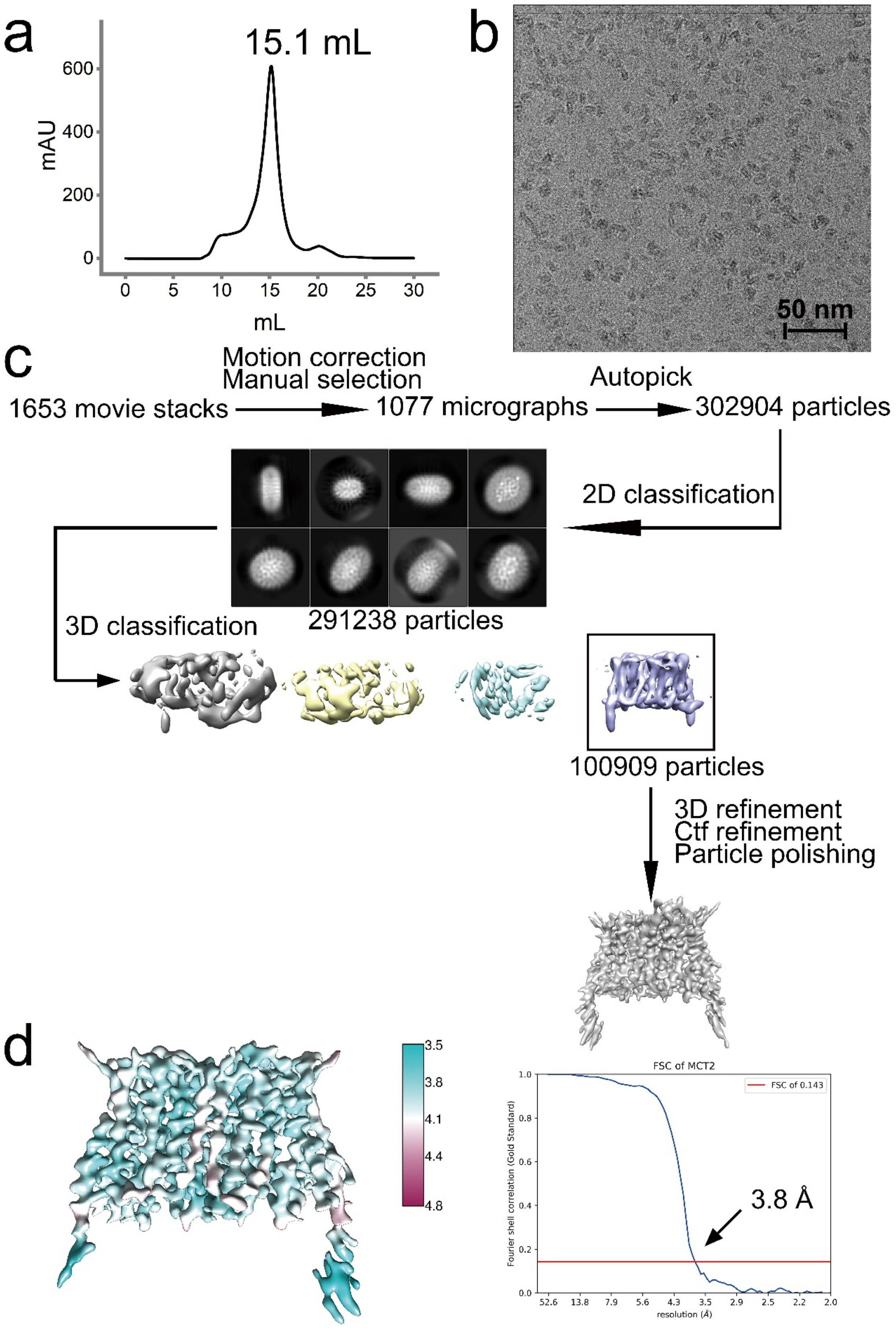

Supplementary Figure 3 | Structure determination of MCT2. a, Size exclusion chromatography of MCT2 on Superpose 6 (GE Healthcare). **b**, Representative cryo-

EM micrograph of MCT2. Scale bar, 50 nm. **c**, Flowchart of image processing for MCT2 particles. **d,** The density maps of MCT2 colored by local resolution and Gold standard FSC curves of the final 3D reconstruction.

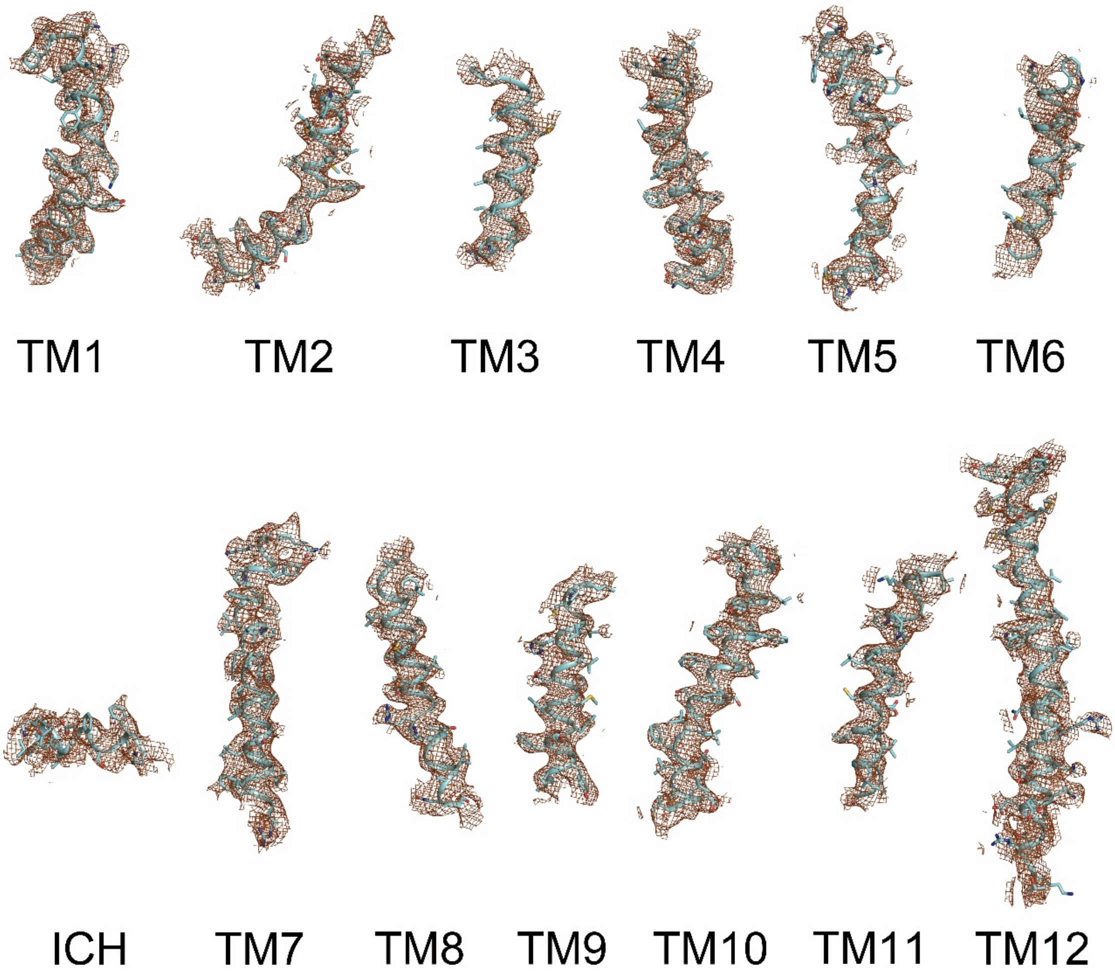

Supplementary Figure 4 | EM density maps of MCT2. Sample maps at 12 transmembrane helices and intracellular helix of MCT2. The maps are low-pass filtered to 3.8 Å and sharpened with a temperature factor of -158.6 Å^2.

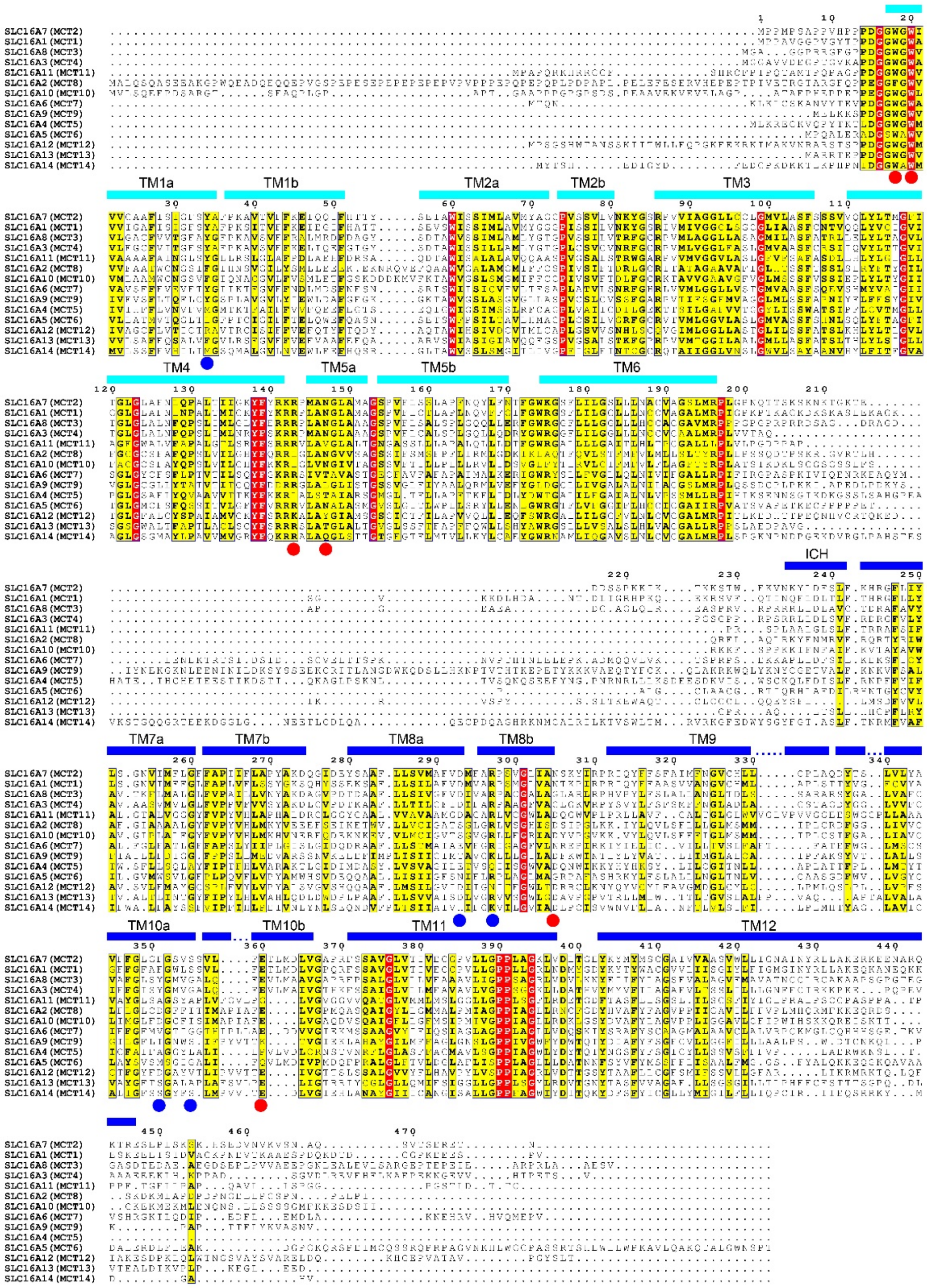

Supplementary Figure 5 | Sequence alignment of human SLC16 members.

Secondary structure assignments are based on the human MCT2 structure. Five

residues for substrate binding are indicated by blue circulars respectively. Red circles mark the residues that participate in polar interactions between the dimer interface.

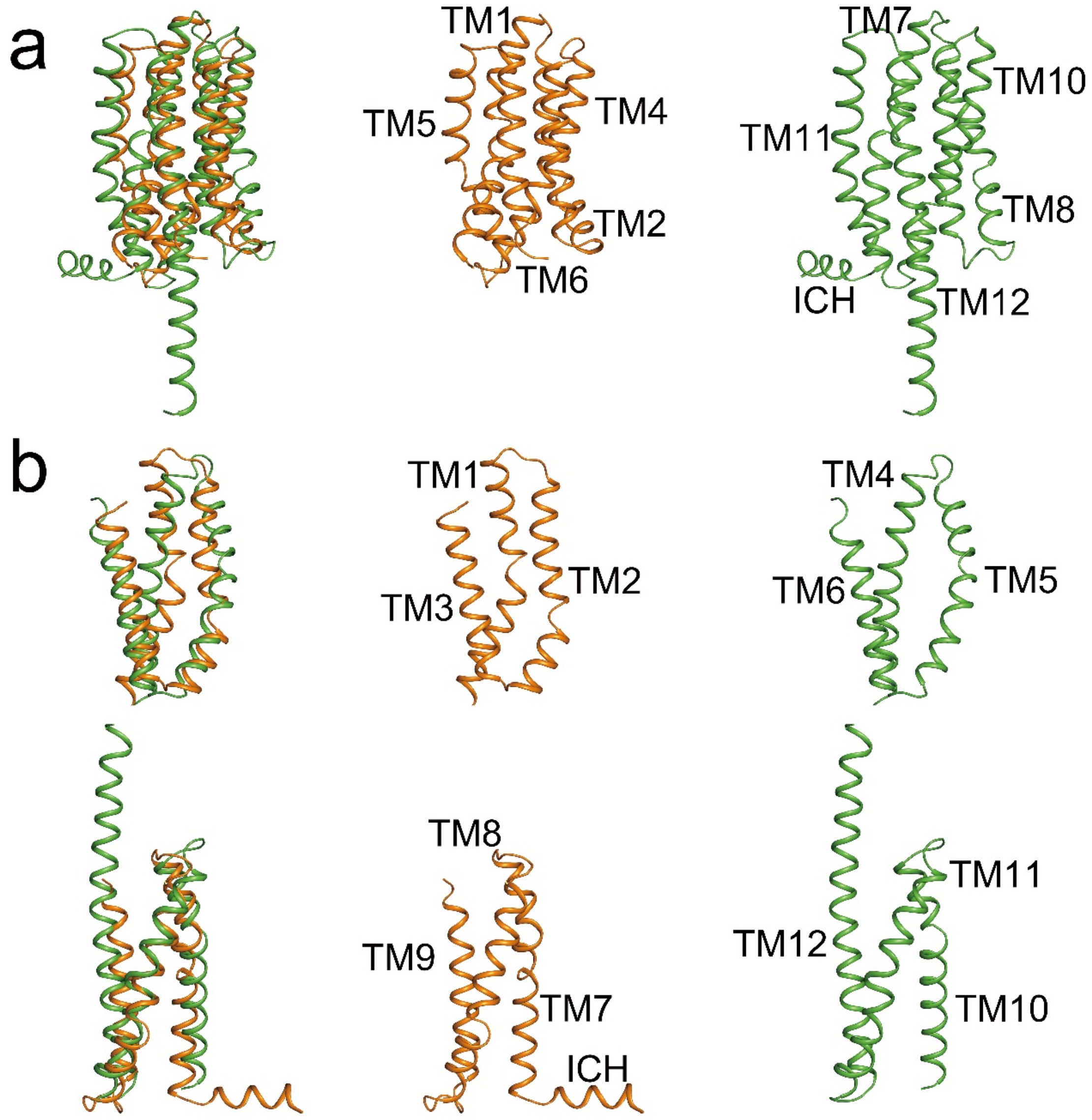

Supplementary Figure 6 | The inverted structural repeat in MCT2. a, Structures of TM1-6 (orange) and TM7-12 (green) shown together or individually in the same orientation. **b,** Structures of TM1-3 (orange) and TM4-6 (green), and TM7-9 (orange) and TM10-12 (green) shown together or individually in the same orientation.

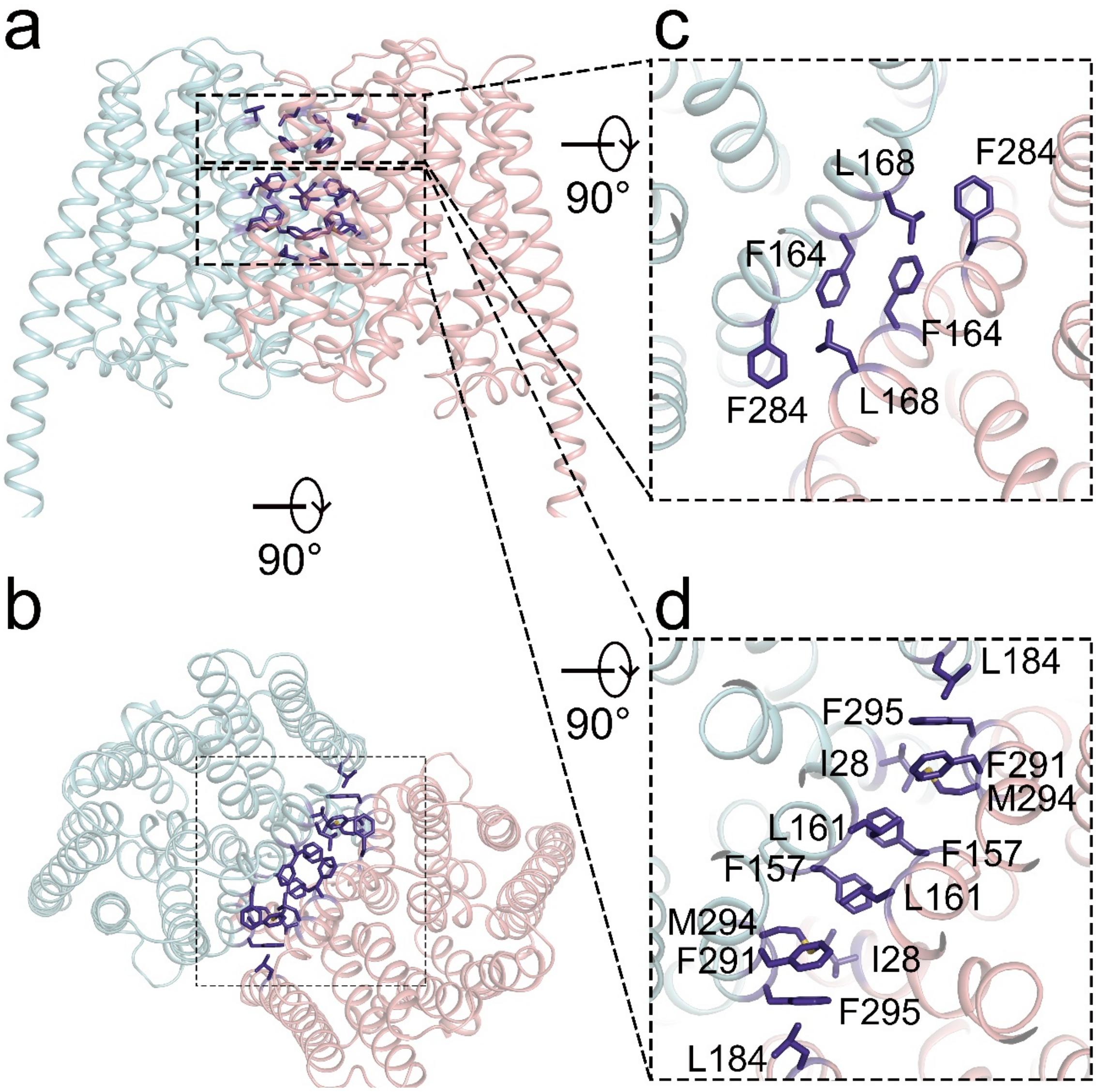

Supplementary Figure 7 | The extensive dimer interface of MCT2. a-b, Cartoon diagrams of MCT2 dimer with the interfacial residues shown **a,** in the same orientation as that in Fig. 2b and **b,** in 90° rotation. **c-d,** zoom in views of the interdimer interactions of two layers.

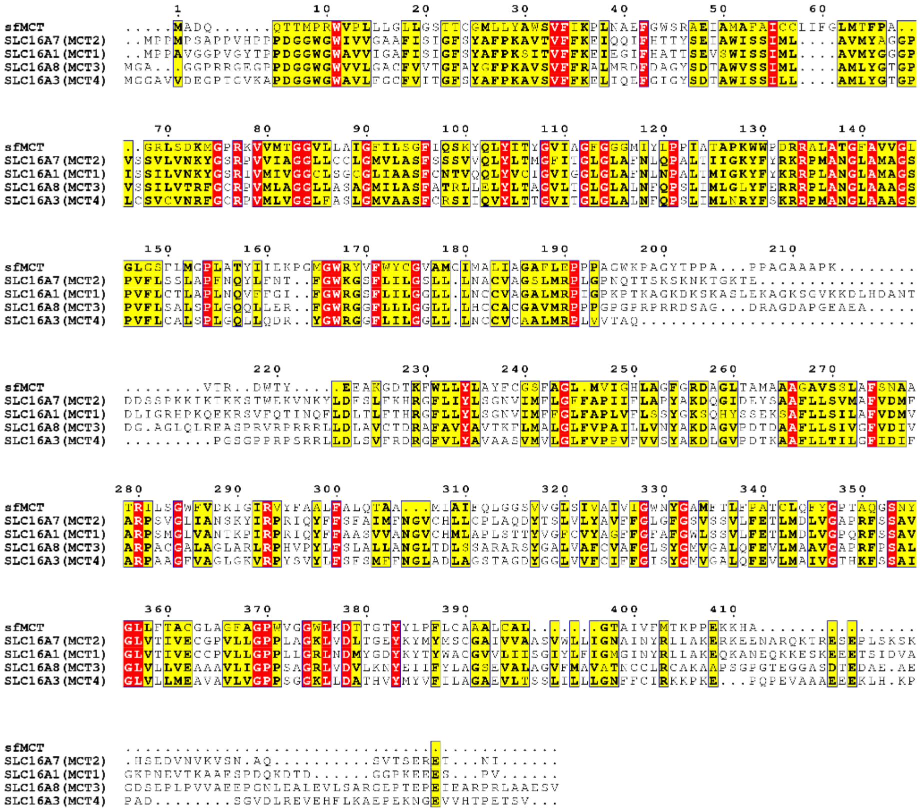

Supplementary Figure 8 | Sequence alignment of SfMCT and human MCTs.

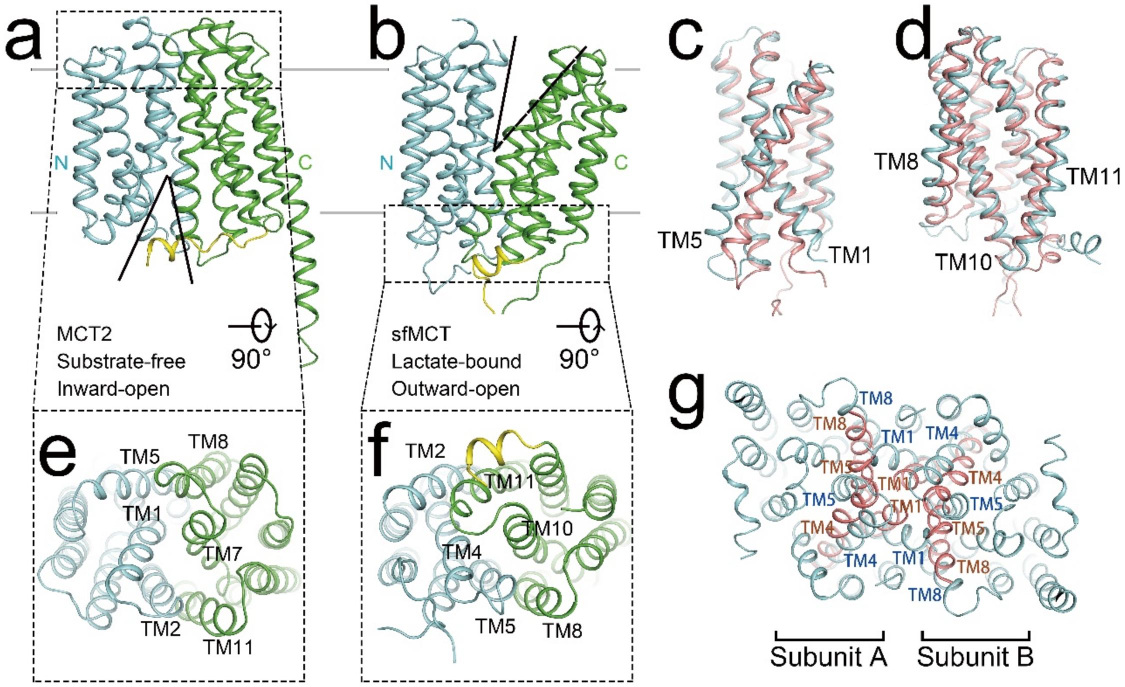

Supplementary Figure 9 | Structural comparison of human MCT2 with its bacterial homologue SfMCT. a-b, Structural comparison of **a,** substrate-free, inward-open MCT2 with **b,** lactate-bound, outward-open SfMCT (PDB ID: 6HCL). N-terminal domain is shown in cyan, whereas C-terminal domain in green. **c-d,** Intra-domain rearrangements of the **c,** N- and **d,** C-terminal domains between the inward-open MCT2 (cyan) and and outward-open SfMCT (orange). **e-f,** Extracellular and intracellular gates observed in **e,** MCT2 and **f,** SfMCT. N- and C- terminal domains are colored as in **a,** and **b**. **g,** Structural comparison of the inward-open structure (cyan) and the outward-open model (orange) of MCT dimer.

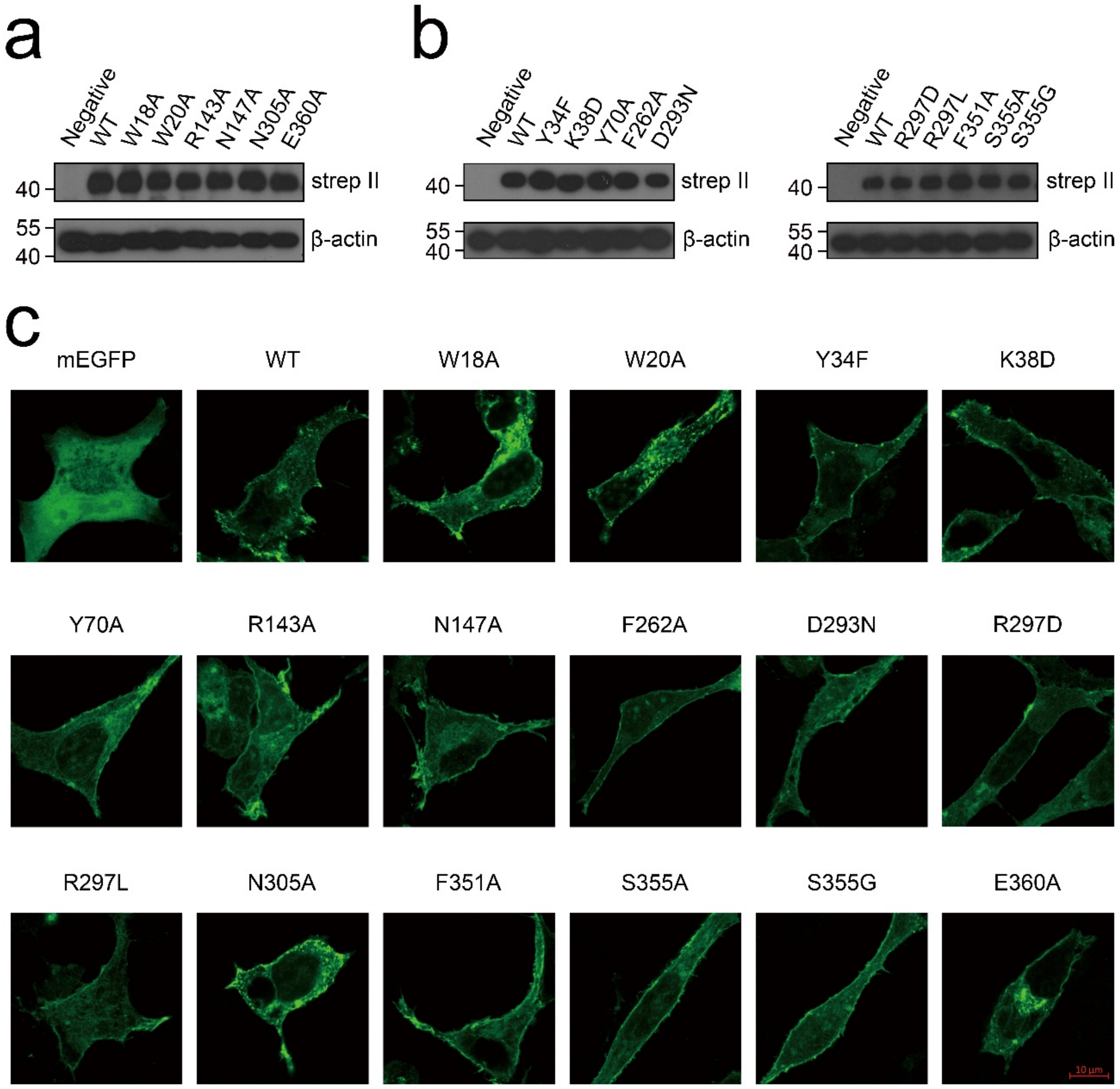

Supplementary Figure 10 | Western blot analysis and cell surface expression of wild type MCT2 and its variants. a-b, Western blot analysis of cellular lysates from HEK293T cells expressing wild type MCT2 and its variants. **c,** Confocal images of localization of mEGFP tagged MCT2 and its variants in HEK293 cells. Scale bar, 10 μm.

Supplementary Table 1 | Data collection and refinement statistics.

**Data collection and processing**	
Magnification	60345.8
Voltage (kV)	300
Electron exposure (e^{-}/Å^2)	~81
Defocus range (μm)	-1.5 to -2.5
Pixel size (Å)	0.41 (Super–resolution mode)
Symmetry imposed	*C2*
Initial particle images (no.)	1,653
Final particle images (no.)	1,077
Map resolution (Å)	3.8
FSC threshold	0.143
**Refinement**	
Model resolution (Å)	3.8
Map sharpening *B* factor (Å^2)	-158.6
Model composition	
Non-hydrogen atoms	6080
Protein residues	788
Ligands	0
r.m.s. deviation	
Bond lengths (Å)	0.007
Bond angles (°)	1.047
Validation	
MolProbity score	1.81
Clashscore	6.34
Rotamer outliers (%)	0.00
Ramachandran plot	
Favored (%)	92.56
Allowed (%)	7.44
Outliers (%)	0

Supplementary Table 2 | Primers used in the study.

Name	Sequences(5'-3')
Strep-F	tcgagggcggaagcagcggcggatggagccacccccagttcgaaaagtgag
Strep-R	tcgactcacttttcgaactgggggtggctccatccgccgctgcttccgccc
MCT2-F	ctagctagcatgccaccaatgccaagtgcccca
MCT2-R	ccgctcgagtaacctcagaaagagaaactaacatt
W18A-F	ccacctccagatggaggagcgggttggattgtgg
W18A-R	ccacaatccaacccgctcctccatctggaggtgg
W20A-F	ctgctccaaccacaatcgcaccccatcctccatctg
W20A-R	cagatggaggatggggtgcgattgtggttggagcag
Y34F-F	tttatctccattggattttccgctgcattccccaaagctgtcac
Y34F-R	gtgacagctttggggaatgcagcggaaaatccaatggagataaa
K38D-F	ttgaagaatacggtgacagcatcggggaatgcataggaaaatc
K38D-R	gattttcctatgcattccccgatgctgtcaccgtattcttcaa
Y70A-F	cttacaggacctcctgcggccataacagccagcataatg
Y70A-R	cattatgctggctgttatggccgcaggaggtcctgtaag
R143A-F	ccaatccatttgccatgggtgccttcctatagaagtatttgc
R143A-R	gcaaatacttctataggaaggcacccatggcaaatggattgg
N305A-F	cgaggtcgaatatatttggaggctgcaattaatcctacagaaggcc
N305A-R	ggccttctgtaggattaattgcagcctccaaatatattcgacctcg
E360A-F	cgaggtccatgagagttgcaaagagaacactgcta
E360A-R	tagcagtgttctctttgcaactctcatggacctcg
F262A-F	agaatataatgggggcagcaaaacctaggaacataatgacatttccagac
F262A-R	gtctggaaatgtcattatgttcctaggttttgctgcccccattatattct
D293N-F	gaaggcctagcaaacatattaacgaaagccataacagatag
D293N-R	ctatctgttatggctttcgttaatatgtttgctaggccttctg
R297D-F	agtttgcaattaatcctacagaaggatcagcaaacatatcaacgaaagccataaca
R297D-R	tgttatggctttcgttgatatgtttgctgatccttctgtaggattaattgcaaact
R297L-F	tgttatggctttcgttgatatgtttgctctaccttctgtaggattaattgcaaact

R297L-R	agtttgcaattaatcctacagaaggtagagcaaacatatcaacgaaagccataaca
F351A-F	ggtattatatgctgtattttttggccttggagctgggagtgttagcagt
F351A-R	actgctaacactcccagctccaaggccaaaaaatacagcatataatacc
S355A-F	caaagagaacactggcaacactcccaaatccaaggccaaaa
S355A-R	ttttggccttggatttgggagtgttgccagtgttctctttg
S355G-F	ttcaaagagaacactgccaacactcccaaatccaagg
S355G-R	ccttggatttgggagtgttggcagtgttctctttgaa
HR-F	ggtccgaagcgcgcggaattcgccaccatgccaccaatgccaagtgc
HR-R	aaacagcacctccaggtcgacaatgttagtttctctttctgaggttacac
atgmEGFP-F	cgcccttagacacagccatggtggcggggccctgaaac
atgmEGFP-R	gtttcagggccccgccaccatggctgtgtctaagggcg
