# Supplementary material for: Cooperative transport mechanism of human monocarboxylate transporter 2
Source: Nat Commun. 2020 May 15;11:2429. doi: 10.1038/s41467-020-16334-1 (PMC7228944; doi:10.1038/s41467-020-16334-1)
Supplement: Supplementary file 3 — Description of Additional Supplementary Files [file 41467_2020_16334_MOESM3_ESM.pdf]

Description of Additional Supplementary Files

File Name: Supplementary Movie 1
Description: Video of pyruvate flux in HEK293T cells expressing MCT2 and pyronic. Time-lapse movie of pyronic fluorescence spectrum fluctuations in the cell shown in Fig. 1a. Frame rate = 1 frame/5s. Cells were initially bathed in an imaging solution without pyruvate, in which the emission intensity at 492 nm (mTFP) was low, but high at 528 nm (Venus). At the time point of 80s, pyruvate influx was initiated by switching the buffer in the perfusion tube in a gravity-driven system to the imaging solution containing 0.4 mM pyruvate. The emission intensity at 492 nm (mTFP) became higher, but lower at 528 nm (Venus). See Extented Data Fig. 1a, and 1b for the spectral images and emission spectrals at selected time frames. At the time point of 240 sec, pyruvate outflux was induced by changing to an imaging solution without pyruvate.

File Name: Supplementary Movie 2
Description: Video of pyruvate flux in HEK293T cells expressing MCT2 and pyronic. Time-lapse movie of pyronic fluorescence spectrum fluctuations in the cell shown in Fig. 1E. Frame rate = 1 frame/2.5s. Cells were initially bathed in an imaging solution containing 10 mM pyruvate. At the time point of 60s, pyruvate efflux was induced by switching the buffer without pyruvate in the perfusion tube in a gravity-driven system to the imaging solution. See Supplementary Fig. 2a and 2b for the spectral images and emission spectrals at selected time frames.
