# Supplementary material for: Cooperative transport mechanism of human monocarboxylate transporter 2
Source: Nat Commun. 2020 May 15;11:2429. doi: 10.1038/s41467-020-16334-1 (PMC7228944; doi:10.1038/s41467-020-16334-1)
Supplement: Supplementary file 6 — Reporting Summary [file 41467_2020_16334_MOESM6_ESM.pdf]

natureresearch

Corresponding author(s): Sheng Ye

Last updated by author(s): 3/31/2020

Reporting Summary

Nature Research wishes to improve the reproducibility of the work that we publish. This form provides structure for consistency and transparency in reporting. For further information on Nature Research policies, see Authors & Referees and the Editorial Policy Checklist .

Statistics

For all statistical analyses, confirm that the following items are present in the figure legend, table legend, main text, or Methods section.

n/a	Confirmed	
☐	☒	The exact sample size (n) for each experimental group/condition, given as a discrete number and unit of measurement
☐	☒	A statement on whether measurements were taken from distinct samples or whether the same sample was measured repeatedly
☐	☒	The statistical test(s) used AND whether they are one- or two-sided<br>*Only common tests should be described solely by name; describe more complex techniques in the Methods section.*
☒	☐	A description of all covariates tested
☒	☐	A description of any assumptions or corrections, such as tests of normality and adjustment for multiple comparisons
☐	☒	A full description of the statistical parameters including central tendency (e.g. means) or other basic estimates (e.g. regression coefficient) AND variation (e.g. standard deviation) or associated estimates of uncertainty (e.g. confidence intervals)
☒	☐	For null hypothesis testing, the test statistic (e.g. F, t, r) with confidence intervals, effect sizes, degrees of freedom and P value noted<br>*Give P values as exact values whenever suitable.*
☒	☐	For Bayesian analysis, information on the choice of priors and Markov chain Monte Carlo settings
☒	☐	For hierarchical and complex designs, identification of the appropriate level for tests and full reporting of outcomes
☐	☒	Estimates of effect sizes (e.g. Cohen's d, Pearson's r), indicating how they were calculated

Our web collection on statistics for biologists contains articles on many of the points above.

Software and code

Policy information about availability of computer code

Data collection	Cryo-EM data collection:DigitalMicrograph(Version2.33.1083.0), SerialEM(Version 3.6.15)
Data analysis	Cryo-EM image analysis software:EMAN2, Gctf, Gautomatch, Relion3.0 beta, Cryosparc2, CisTEM, MotionCor2,Coot, Pymol, Phenix, MolProbity, UCSF Chimera<br>Pyruvate transport activity analysis softwares:Igor Pro software (Version 5.05, WaveMetrics), OriginPro 2019 (OriginLab)

For manuscripts utilizing custom algorithms or software that are central to the research but not yet described in published literature, software must be made available to editors/reviewers. We strongly encourage code deposition in a community repository (e.g. GitHub). See the Nature Research guidelines for submitting code & software for further information.

Data

Policy information about availability of data

All manuscripts must include a data availability statement. This statement should provide the following information, where applicable:
- Accession codes, unique identifiers, or web links for publicly available datasets
- A list of figures that have associated raw data
- A description of any restrictions on data availability

Provide your data availability statement here.

Field-specific reporting

Please select the one below that is the best fit for your research. If you are not sure, read the appropriate sections before making your selection.

- [x] Life sciences
- [ ] Behavioural & social sciences
- [ ] Ecological, evolutionary & environmental sciences

For a reference copy of the document with all sections, see nature.com/documents/nr-reporting-summary-flat.pdf

Life sciences study design

All studies must disclose on these points even when the disclosure is negative.

Sample size	All living cell imaging data were repeated at least three times with different cells. The sample size is determined based on the reproducibility of the data.
Data exclusions	For living cell imaging data, obvious outliers and data with poor signal to noise ratio were excluded.<br>For cryo-EM data, 3-D classification yielded multiple 3-D reconstruction maps. Only the particles that gave rise to homogeneous density were selected, combined and used in the final reconstruction and refinement. Details are described in the flowchart of Extended Data Figure 3 and Methods.
Replication	All experiments were repeated at least 3 times with similar results.
Randomization	For living cell imaging, cells with fluorescence (cells expressing pyronic) were randomly selected for imaging.
Blinding	The investigators were not blinded to group allocation.

Reporting for specific materials, systems and methods

We require information from authors about some types of materials, experimental systems and methods used in many studies. Here, indicate whether each material, system or method listed is relevant to your study. If you are not sure if a list item applies to your research, read the appropriate section before selecting a response.

Materials & experimental systems

n/a	Involved in the study	
☐	☒	Antibodies
☐	☒	Eukaryotic cell lines
☒	☐	Palaeontology
☒	☐	Animals and other organisms
☒	☐	Human research participants
☒	☐	Clinical data

Methods

n/a	Involved in the study	
☒	☐	ChIP-seq
☒	☐	Flow cytometry
☒	☐	MRI-based neuroimaging

Antibodies

Antibodies used	Rabbit anti-strep-tag II (Abacm; ab76949; 1:4000 dilution). Supplementary Figure 10a-b.<br>Mouse anti-β-actin (Huabio; M1210-2; 1:5000 dilution). Supplementary Figure 10a-b.<br>Goat anti-rabbit immunoglobulin G (Sangon Biotech; D110058; 1:2000 dilution). Supplementary Figure 10a-b.<br>Goat anti- mouse IgG (Sangon Biotech; D110087; 1:2000 dilution). Supplementary Figure 10a-b.
Validation	Rabbit anti-strep-tag II is used for western blot to detect Strep-tagged protein expressed in HEK293 cells. The manufacture's website states the species reactivity is species independent.<br>Mouse anti-β-actin is used for western blot to detect β-actin in HEK293 cells. The manufacture's website states the species reactivity includes human, mouse and rat.

Eukaryotic cell lines

Policy information about cell lines

Cell line source(s)	HEK293F: Thermo Fisher Scientific; R79007<br>HEK293T: ATCC; CRL-3216<br>HEK293: ATCC; CRL-1573<br>Sf9: ATCC; CRL-1711
Authentication	All cell lines from ATCC and Thermo Fisher Scientific were not further authenticated.

Mycoplasma contamination	None have been tested for mycoplasma contamination.
Commonly misidentified lines<br>(See ICLAC register)	No commonly misidentified cell lines were used.
